# *Trypanosoma cruzi* Binds to Cytokeratin through Conserved Peptide Motifs Found in the Laminin-G-Like Domain of the gp85/Trans-sialidase Proteins

**DOI:** 10.1371/journal.pntd.0004099

**Published:** 2015-09-23

**Authors:** Andre Azevedo Reis Teixeira, Veronica de Cássia Sardinha de Vasconcelos, Walter Colli, Maria Júlia Manso Alves, Ricardo José Giordano

**Affiliations:** Department of Biochemistry, Chemistry Institute, Universidade de São Paulo, São Paulo, Brazil; Harvard School of Public Health, UNITED STATES

## Abstract

**Background:**

Chagas' disease, caused by the protozoan parasite *Trypanosoma cruzi*, is a disease that affects millions of people most of them living in South and Central Americas. There are few treatment options for individuals with Chagas' disease making it important to understand the molecular details of parasite infection, so novel therapeutic alternatives may be developed for these patients. Here, we investigate the interaction between host cell intermediate filament proteins and the *T*. *cruzi* gp85 glycoprotein superfamily with hundreds of members that have long been implicated in parasite cell invasion.

**Methodology/Principal Findings:**

An *in silico* analysis was utilized to identify peptide motifs shared by the gp85 *T*. *cruzi* proteins and, using phage display, these selected peptide motifs were screened for their ability to bind to cells. One peptide, named TS9, showed significant cell binding capacity and was selected for further studies. Affinity chromatography, phage display and invasion assays revealed that peptide TS9 binds to cytokeratins and vimentin, and prevents *T*. *cruzi* cell infection. Interestingly, peptide TS9 and a previously identified binding site for intermediate filament proteins are disposed in an antiparallel β-sheet fold, present in a conserved laminin-G-like domain shared by all members of the family. Moreover, peptide TS9 overlaps with an immunodominant T cell epitope.

**Conclusions/Significance:**

Taken together, the present study reinforces previous results from our group implicating the gp85 superfamily of glycoproteins and the intermediate filament proteins cytokeratin and vimentin in the parasite infection process. It also suggests an important role in parasite biology for the conserved laminin-G-like domain, present in all members of this large family of cell surface proteins.

## Author Summary

Chagas' disease affects millions of people worldwide and is caused by a microorganism called *Trypanosoma cruzi*. Treatment options for patients with Chagas' disease is still limited to a small number of drugs, all of them very toxic with important side effects that can be debilitating for the health of patients. Understanding the molecular details of how *T*. *cruzi* infects humans is an important step toward the development of new drugs for this disease. As part of its life cycle, *T*. *cruzi* has to invade cells in order to replicate and produce new parasites. This is a complex event, which involves different proteins produced by both the parasite and the human host cells. Among them, there is a large family of highly polymorphic *T*. *cruzi* proteins important to guide the parasite to the target cells. Here we show that notwithstanding their differences, all members of this family share a small region comprised of nine amino acids that is important for cell recognition and infection by the parasite. Exploring these findings may provide researchers with new insights on how to prevent *T*. *cruzi* cell invasion and lead to novel therapeutic alternative for this debilitating disease.

## Introduction

Chagas’ disease, also known as American trypanosomiasis, is a tropical and neglected disease caused by the parasite *Trypanosoma cruzi*. The disease affects 7 to 8 million people worldwide most of them living in South and Central American countries, accordingly to recent assessments from the World Health Organization, (http://www.who.int/mediacentre/factsheets/fs340/en/). However, migration has spread the disease to all countries in the Americas and several European and Western Pacific countries. In endemic areas, the parasite is transmitted to humans primarily by triatomine insect vectors (kissing bug) while in non-endemic countries, transmission is restricted by contact with contaminated blood or tissues, such as blood transfusions and organ transplants [1]. Chagas’ disease can be a life-threatening and debilitating illness to those that develop cardiomyopathy or one of the digestive forms of the disease making it a significant burden for health care and society at large [2].

Treatment options for patients with Chagas’ disease is still limited to a small number of drugs, all of them very toxic with important side effects that can be debilitating for the health of patients. These drugs are most effective if administered during the acute phase of the disease, a narrow period that lasts for a few weeks soon after infection [3]. But because infected individuals are often asymptomatic or experience only mild fever during the initial stages of the disease, it is difficult for physicians to diagnose correctly Chagas’ disease until it is too late for treatment. In addition to these limitations, there have already been several documented cases of *T*. *cruzi* strains resistant to currently available drugs [3,4]. It is therefore urgent to develop therapeutic alternatives for Chagas’ disease. To understand the molecular details on how *T*. *cruzi* interacts with host-cell proteins is an important step toward this goal and to the development of novel therapeutic agents for the disease.

To tackle these challenges, our group has been studying a large family of surface glycoproteins firstly described in our laboratory, which has been implicated in cell adhesion and invasion by *T*. *cruzi* [5,6]. This protein was initially identified as an 85-kDa surface protein expressed selectively by the infective forms of the parasite and named Tc-85 [5,7]. Today we know that this protein belongs to a large family of proteins encoded by multiple genes, which are collectively known as the gp85/trans-sialidase (gp85/TS) multigene family [6,8–10]. With hundreds of genes in the genome, all gp85/TS proteins share in common a sialidase domain with Asp-boxes motifs and the peptide motif VTVxNVxLYNRPLN [8]. Based on sequence similarity, the proteins encoded by this gene family were further subdivided in eight groups [11]. Proteins that clustered in group I are the only proteins that have enzymatically active trans-sialidase domains, which can remove sialic acid residues from glycoproteins present on the mammalian cell surface and transfer it to glycoproteins on the parasite cell membrane. It has been claimed that the trans-sialidase activity is essential for parasite invasion [12].

The Tc-85 proteins belong to the group II of the gp85/TS family but lack enzymatic activity and cannot transfer sialic acid. Instead, they have been implicated in cell adhesion and parasite cell entry [10]. They interact with different receptors present in the extracellular matrix and cell surface, such as laminin [13–15], the intermediate filament proteins cytokeratin and vimentin [16,17], fibronectin [18], mucin [19] and the prokineticin-2 receptor [20]. In earlier studies, we showed that the peptide VTVTNVFLYNRPLN (denominated FLY for short, and derived from the VTVxNVxLYNRPLN motif) present in Tc-85 is a cytokeratin binding site important for parasite cell adhesion and invasion [16]. Binding of Tc-85 to cytokeratin activates ERK1/2 signaling cascade, resulting in an increase in the number of parasites per cell [21]. Also, using phage display as a surrogate for the peptide, we showed that the peptide FLY may contribute to tissue tropism by targeting the parasite to specific vascular beds [17]. Taken together, our studies suggest an important role for cytokeratins and the peptide FLY present in proteins from the gp85/TS family in parasite cell adhesion and invasion.

Herein, we expanded these studies to explore other common peptide motifs found in the gp85/TS proteins in order to check for their possible role in parasite cell adhesion and invasion. By sequence similarity of proteins belonging to the group II, we identified several peptide conserved among all members of this family. Using peptide phage display, these peptides were expressed in bacteriophage and analyzed for their capacity to bind to cells. The results have shown that one of the selected peptides bind to cells and inhibits parasite cell invasion. Interestingly, this peptide also binds to cytokeratin and vimentin, and is found in the three dimensional structure of the protein side by side with the peptide FLY comprising a β-anti-parallel fold that is part of the Laminin-G-like domain (LamG) present in all gp85/TS family members. Therefore, the results herein reported provide further evidence supporting the claim that *T*. *cruzi* interacts with intermediate filament proteins, with the LamG domain of the gp85/TS family members being an important element in parasite cell adhesion and invasion.

## Results

### Identification of conserved peptides in gp85/trans-sialidase group II proteins

**Fig 1:**
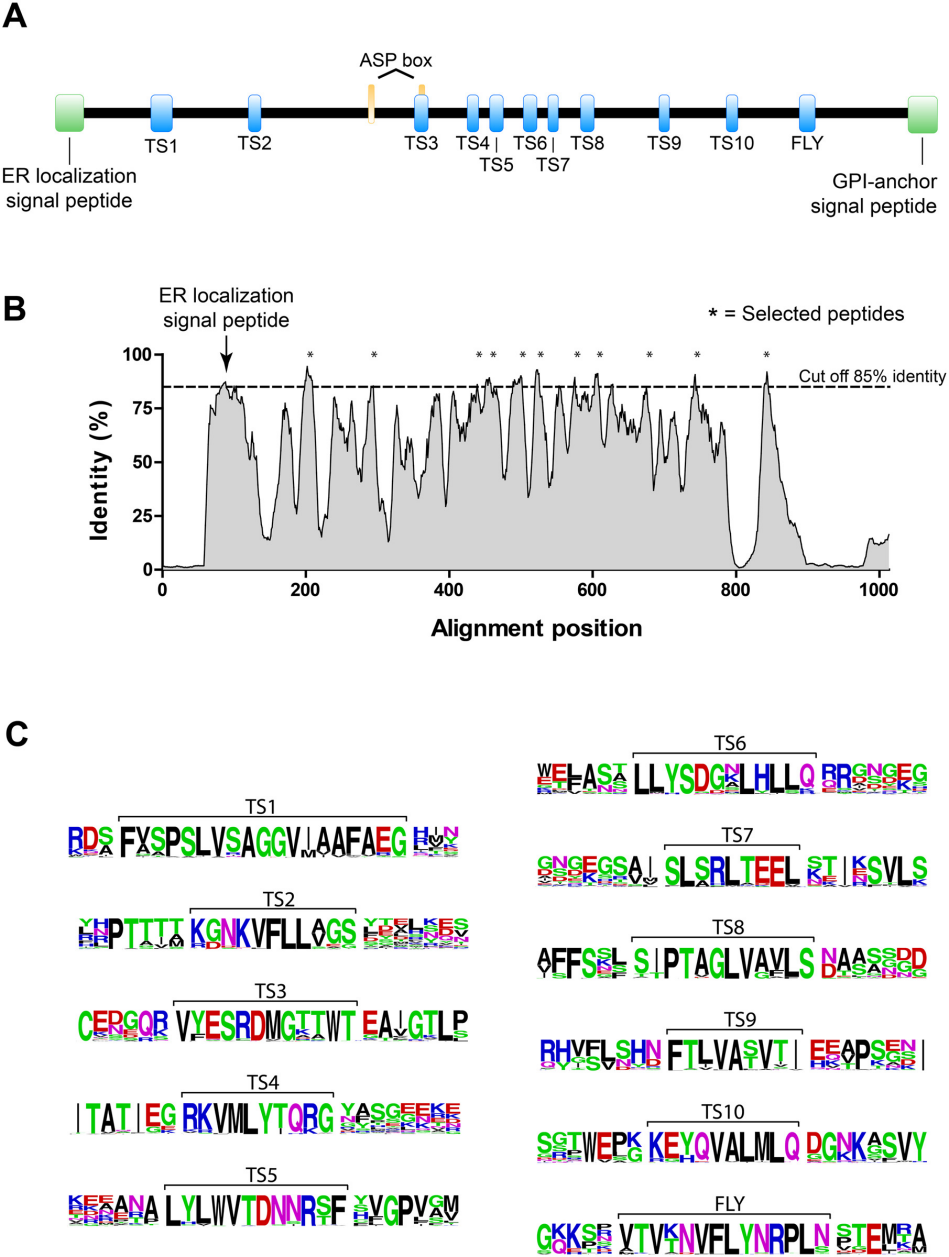
Selection of peptides common to all members of the gp85/trans-sialidase family. **(A)** General representation of gp85/TS proteins belonging to the group II. The signal peptides for endoplasmic reticulum (ER) localization and glycophosphatidylinositol (GPI) anchor (green boxes) are indicated; the sialidase ASP-box repeats (orange boxes) and the conserved peptides identified in this study (blue boxes), which include the VTVxNVxLYNRLN motif denominated FLY, are shown. **(B)** Moving average of nine amino acids for each position in the gp85/TS group II protein alignment. The signal peptide for ER localization (arrow) and the 10 most conserved peptides (*) are indicated. **(C)** Representation in sequence logo format of the gp85/TS derived peptides (shown within brackets). The letter size indicates amino acid conservation in each position and the color, whether amino acids are polar (green), hydrophobic (black), positively (blue) or negatively (red) charged.

To identify conserved peptide motifs shared by members of the group II of the gp85/TS family, we aligned 117 protein sequences belonging to this family. Individual sequences were obtained from the TryTryp database [22] and considered as belonging to the group II of the gp85/TS family accordingly to a previously described classification [11]. As expected, analysis of the primary sequences showed that most proteins from group II have molecular mass ranging from 80 to 90 kDa and contain the ASP-box and the VTVxNVxLYNRPLN motifs common to all members of the family (Fig 1A). To visualize and quantify the results of our alignment, first we generated a grid profile to express amino acid frequencies for each position in the alignment (S1 Table). Next, the moving average for each position was calculated by averaging the frequencies for the initial position plus the frequency of the following eight amino acids. This was done to minimize fluctuations and to facilitate the visualization of conserved regions in the gp85/TS family members (Fig 1B). We chose a moving average of 9 amino acids because it approximates the peptide length that can be displayed by the bacteriophage (see below). Moreover, similar analysis performed with moving averages corresponding to peptides of 7 up to 12 amino acids produced similar profiles. Based on these results, a cut off of 85% identity was chosen to select the 10 most conserved peptides. The selected peptides ranged from 9 to 19 amino acids in length with average identities varying from 85.5 to 92.1% (Fig 1C). The selected peptides were denominated TS1 to TS10 (Table 1 and S2 Table). It is important to notice that almost all analyzed proteins have an amino-terminal signal peptide, which is not found in the mature protein, and therefore these regions were not considered for peptide selection (Fig 1B, **arrow**).

**Table 1:** The gp85/TS derived peptides grafted into phage.

Name	Sequence
**TS1a**	SPSLVSAGGVIAAFAE
**TS1b**	FVSPSLVSAGGV
**TS2**	KGNKVFLLAGS
**TS3**	VYESRDMGTTWT
**TS4**	RKVMLYTQRG
**TS5**	LYLWVTDNNRSF
**TS6**	LLYSDGNLHLLQ
**TS7**	SLSRLTEEL
**TS8**	SIPTAGLVAVLS
**TS9**	FTLVASVTI
**TS10**	KEYQVALMLQ
**FLY**	VTVTNVFLYNRPLN

### Peptide TS9 binds to cells

**Fig 2:**
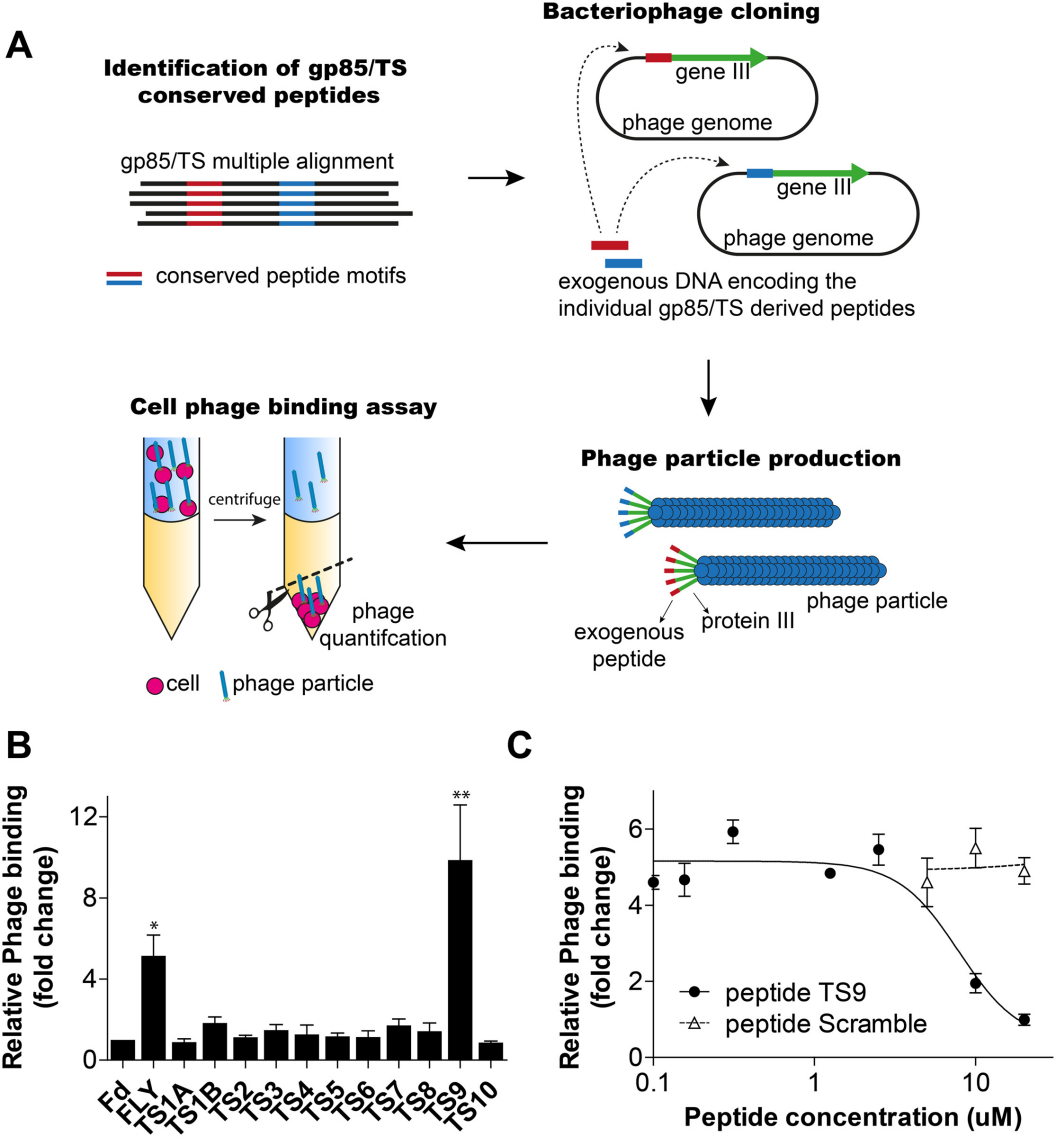
Screening of peptides by phage display. **(A)** Flowchart summarizing the pipeline for *in silico* selection, bacteriophage display and the *in vitro* functional assay (BRASIL method) for the identification of conserved peptides derived from the gp85/TS family with cell binding properties. **(B)** Quantification of phage binding to LLC-MK_2_ cells using the BRASIL methodology and quantitative PCR (qPCR). Phage binding results were normalized relative to the control phage Fd-tet (insertless phage). Mean ± standard error of the means (SEM) from four samples are shown. Significant cell binding was observed for phage FLY and TS9 (* p<0.05 and *** p<0.001; one way ANOVA, N = 4). **(C)** Peptide competition assay. Effect of synthetic peptide TS9 and the control scramble peptide in phage TS9 binding to LLC-MK_2_. Phage binding results were normalized relative to the control phage Fd-tet. Mean ± SEM of a representative experiment performed in triplicate are shown.

Having identified common peptide motifs present in all gp85/TS family members, we next sought to determine whether they bind to cells. For that, we employed the phage display technique, which uses bacteriophage as a surrogate for the peptide [17,23]. In this assay, the phage genome is engineered to allow the production of phage particles that expressed each individual TS peptide fused to the capsid protein III (pIII) of the bacteriophage (Fig 2A). Phage binding was then performed using the BRASIL method, which allows for separation and quantification of cells with their respective bound phage in a single centrifugation step [23].

All selected peptides were successfully displayed in the bacteriophage with the exception of peptide TS1. The latter is the longest peptide with 19 amino acids, being too large for pIII display. As is known, the pIII is involved in phage infection and it is often inactivated by peptides longer than 15 amino acids. To solve this problem, peptide TS1 was split into two shorter peptides named TS1a and TS1b, which were successfully displayed by the bacteriophage. These peptides were 16 and 12 amino acids in length, respectively, and contained a 10 amino acid overlap in sequence to increase the likelihood of preserving a potential cell binding epitope present within the original TS1 peptide (Table 1).

Next, the epithelial cell line LLC-MK_2_ was selected for the cell binding assay since this cell line is routinely utilized for *T*. *cruzi* culture and infection studies [5,7]. Among all phage tested in our binding assay, only phage expressing peptide TS9 bound to LLC-MK_2_ cells consistently in independent experiments (Fig 2B). As expected, no binding was observed with the negative control phage Fd-tet that does not display any exogenous peptide and the positive control phage FLY bound to cells [16,17]. Binding of phage TS9 to LLC-MK_2_ cells was inhibited in a dose dependent manner by the cognate synthetic peptide TS9 (Fig 2C). Phage binding was not affected by the scrambled control peptide. The scrambled peptide used as control has the same amino acid composition of peptide TS9 but with a different primary sequence (sequence LTVIFATVS). These results confirm that phage TS9 binding to cells is mediated by the displayed peptide and due to a random phage mutation. In summary, we have identified a new cell binding peptide present in all members of the gp85/TS family group II.

### The motif including peptide TS9 is conserved in all gp85/trans-sialidases

Our initial analysis to select conserved peptides took in consideration only members that belonged to the group II of the gp85/TS family. To determine whether the cell binding peptide TS9 is present in all members of the family, we aligned 505 protein sequences obtained from the TryTryp database and belonging to all 8 groups of the gp85/TS family. The alignment revealed that the motif containing the peptide TS9 is conserved in all members of the family (S1 Fig). The motif encoded in peptide TS5 is also conserved in all members of the family while some of the other TS peptides are partially conserved in select groups. For instance, the motif encoding peptide TS3 is present in proteins belonging to groups I, II, IV, V and VI. But overall, only peptide motifs TS5, TS9 and FLY are conserved throughout all groups of the family. On the whole, the motifs TS9 and FLY co-occur in all members of the family with the exception of a few truncated members that lacks FLY only or both motifs. In summary, the motif TS9 is highly conserved throughout proteins of all groups of the gp85/TS family.

### Cytokeratin is a receptor for peptide TS9

Having shown that peptide TS9 binds to LLC-MK_2_ cells, a search to identify the cell surface receptor for this peptide was undertaken. To this end, we employed affinity chromatography with the immobilized peptide TS9. Because the peptide is displayed on the bacteriophage fused to the amino-terminal end of the pIII protein, it was immobilized on the resin by its terminal carboxyl group for better mimicking the spatial orientation of the peptide in the bacteriophage. Protein extracts from LLC-MK_2_ cells were then incubated with the immobilized peptide and after extensive wash, bound proteins were eluted sequentially with 1% sodium dodecyl sulfate (SDS) and 8M urea (in phosphate buffer). All eluates were analyzed by protein electrophoresis (SDS-PAGE).

**Fig 3:**
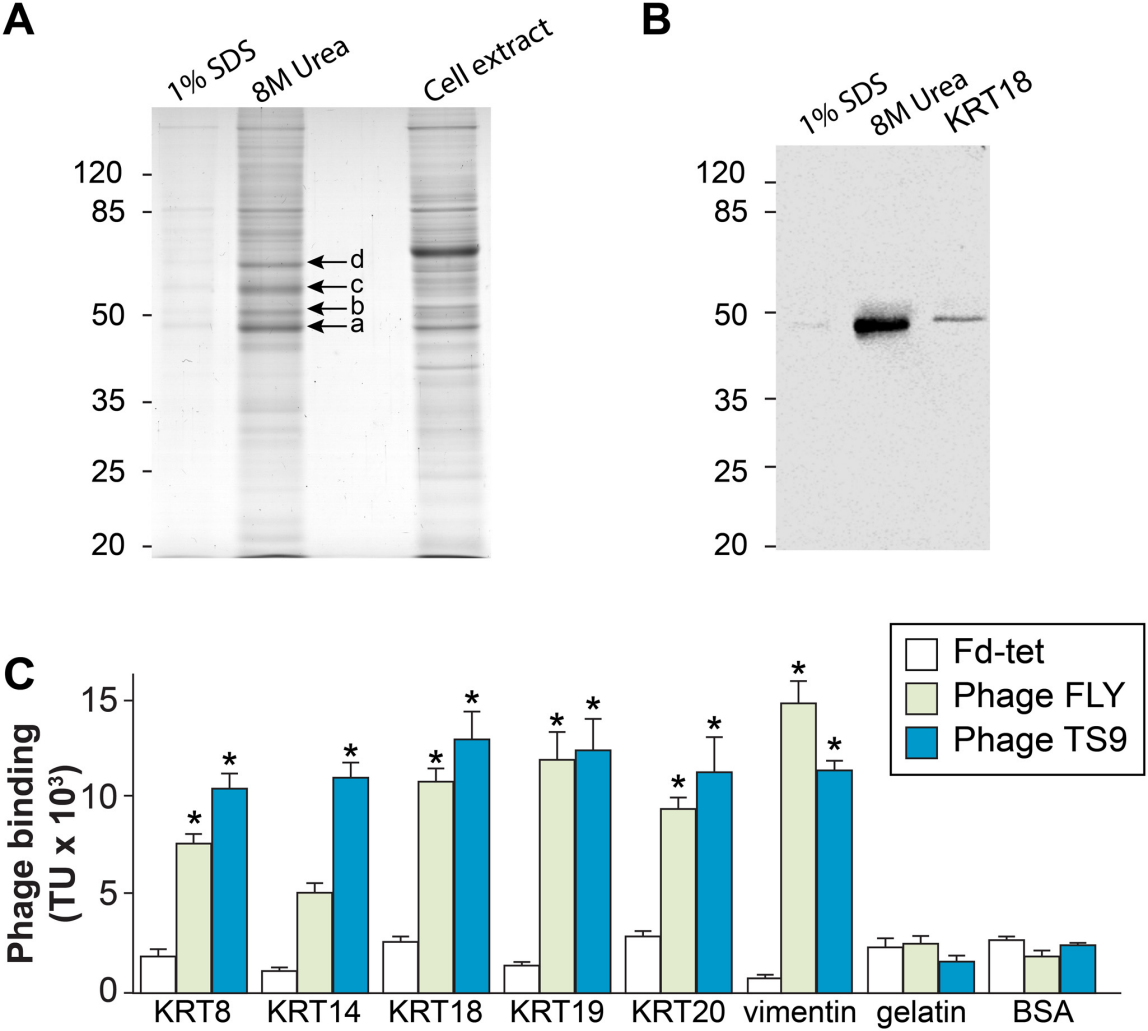
Cytokeratin is the receptor to peptide TS9. **(A and B)**. Affinity chromatography of LLC-MK_2_ cell extract using immobilized peptide TS9, eluted sequentially with 1% SDS and 8M urea, were analyzed by SDS-PAGE and Coomassie staining **(A)** or Western-blot using anti-KRT18 antibody **(B)**. In **(A)**, proteins with calculated masses of 49, 53, 59 and 66 kDa identified by Coomassie staining (arrows a-d, respectively) were excised from the gel and analyzed by mass spectrometry. **(B)** Immunoreactivity of anti-KRT18 antibody with the 1% SDS and 8M urea eluates; recombinant KRT18 was used as positive control. **(C)** Binding of phage TS9, FLY and Fd-tet (insertless phage) to BSA, recombinant human cytokeratins 8, 14, 18, 19 and 20, vimentin or gelatin. Mean ± SEM of a representative experiment performed in triplicate are shown (* indicates p<0.05, two-way ANOVA).

Proteins with calculated molecular masses of 49, 53, 59 and 66 kDa were enriched in the 8M urea eluate, compared with the total extract (Fig 3A). The regions containing these proteins were excised from the gel and analyzed by mass spectrometry for protein identification. Cytokeratin-18 and cytokeratin-8 (KRT18 and KRT8) were the proteins with the highest scores identified as possible ligands for peptide TS9 present in the region of the gel corresponding to molecular masses of 49 and 53 kDa (S3 Table and Fig 3A, proteins indicated by arrows a and b). Interestingly, two other cytoskeletal protein (actin, 49 kDa, arrow a; and tubulin, 59 kDa, arrow c) and HSP90 (66 kDa, arrow d) were identified as a possible ligands for peptide TS9 or more likely, they result from the strong association with the cytoskeleton and associated proteins S3 Table) (Fig 3A). Because KRT18 and KRT8 are both constituents of intermediate filaments and have been previously identified by our group as receptors for the peptide FLY [16], we decided to focus our study on the interaction between peptide TS9 and cytokeratins. Immunoblots further confirmed the presence of KRT18 in the eluate (Fig 3B).

To confirm that peptide TS9 binds to cytokeratin, we employed again a phage display assay. Because there are several genes encoding different cytokeratin proteins, and cells often express more than one gene, we expanded our validation to include other recombinant members of this family (as well as vimentin, another intermediate filament protein). The results of our binding assay show that both phage TS9 and FLY bind to all intermediate filament proteins that were tested (Fig 3C). The interaction is specific since no binding of phage TS9 was observed to gelatin or bovine serum albumin (BSA), and the control phage Fd-tet did not bind to any of the proteins tested. The binding profile of phage TS9 is also in agreement with the results of the affinity chromatography in which at least two different cytokeratin proteins were eluted from the peptide column. In summary, these data corroborate previous reports showing that proteins from the gp85/TS family can interact with different cytokeratins and intermediate filament proteins expressed by cells [16,17,21].

### Peptide TS9 and FLY form a possible binding site in the LamG domain

**Fig 4:**
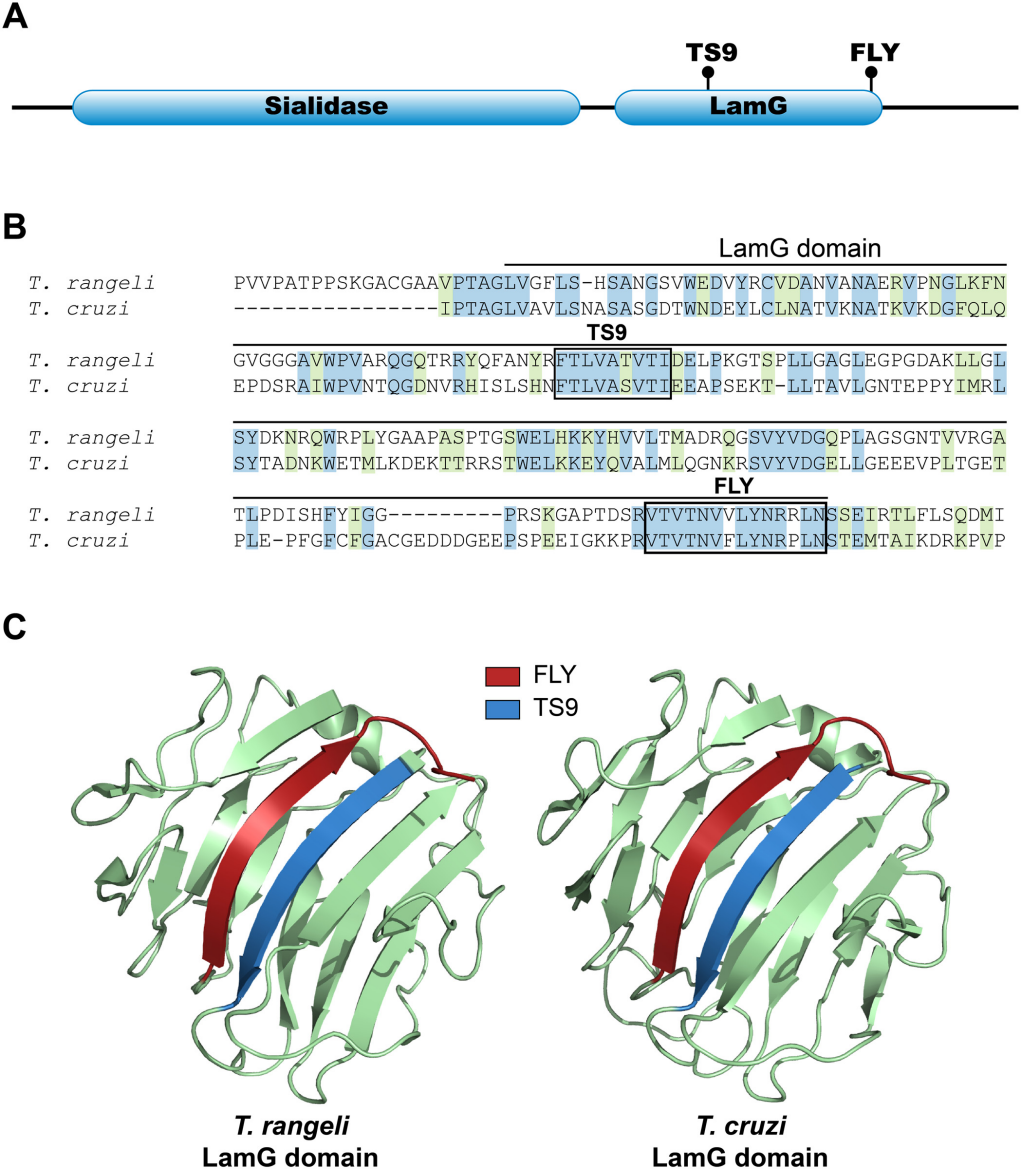
Structural analysis of peptides FLY and TS9. **(A)** Schematic representation of the two conserved domains (Sialidase and LamG) present in the gp85/TS family members. The location of peptides TS9 and FLY are indicated. (B) Sequence alignment of the LamG domain from Tc85-11 and from a *T*. *rangeli* sialidase (sequence from PDB ID 1WCS, amino acids 426–624). Identical amino acids are highlighted in blue and conservative changes in green. Peptides TS9 and FLY are shown (boxes). **(C)** Ribbon diagrams representing the protein structures of the LamG domain from the *T*. *rangeli* sialidase (1WCS) and from the 3D modeling of the *T*. *cruzi* Tc85-11 protein. The positions of peptides TS9 (blue) and FLY (red) are highlighted in the structure.

Both peptides TS9 and FLY are separated from each other by approximately 100 amino acids in the primary sequence of the gp85/TS proteins and, therefore, they cannot form a linear binding site. So, we questioned whether they could be located next to each other in the tridimensional structure of the protein, thus constituting a conformational binding site. In fact, proteins belonging to the gp85/TS family are typically composed of two domains: a sialidase domain at the amino-terminus (NCBI CDD id: cl21531) and a laminin-G like domain (LamG) at the carboxyl-terminus (NCBI CDD id: cl00102). Indeed, both peptides TS9 and FLY are contained within the LamG domain, the peptide TS9 being located towards the beginning of the domain, while FLY is at the carboxyl-terminus (Fig 4A). To assess the locations of peptides TS9 and FLY in the tridimensional structure of the LamG domain, we analyzed available tridimensional structure of trypanosomatid sialidases as well as a molecular model of the LamG domain from a *T*. *cruzi* group II gp85/TS family member. We selected the Tc85-11 protein (GenBank id: AAD13347) as a representative member because it is a cell binding protein belonging to the group II of the gp85/TS family [14] and it was previously used in molecular modeling studies by our group [15]. By searching the Research Collaboratory for Structural Bioinformatics (RCSB) protein databank we identified a close homologue of Tc85-11 with a known tertiary structure of the sialidase from *Trypanosoma rangeli* (PDB id: 1WCS) [24]. This protein shares 50% of sequence similarity with Tc85-11 and contains both peptides TS9 and FLY with substitutions that are also present in other members of *T*. *cruzi* gp85/TS family (Fig 4B). To build the Tc85-11 model we used the I-Tasser server [25] having the 1WCS structure as a template. Analysis of the crystal structure of the sialidase and the Tc85-11 model indicated that, although the peptides TS9 and FLY are further apart in the primary sequence, they are close to each other in space constituting antiparallel β-sheet structures (Fig 4C). These data suggest that peptides TS9 and FLY may comprise a single non-linear conformational binding site for cytokeratins present in all members of the gp85/TS family and further implicates the LamG domain as important for parasite adhesion.

### The gp85/trans-sialidase LamG domain binds to cytokeratins

**Fig 5:**
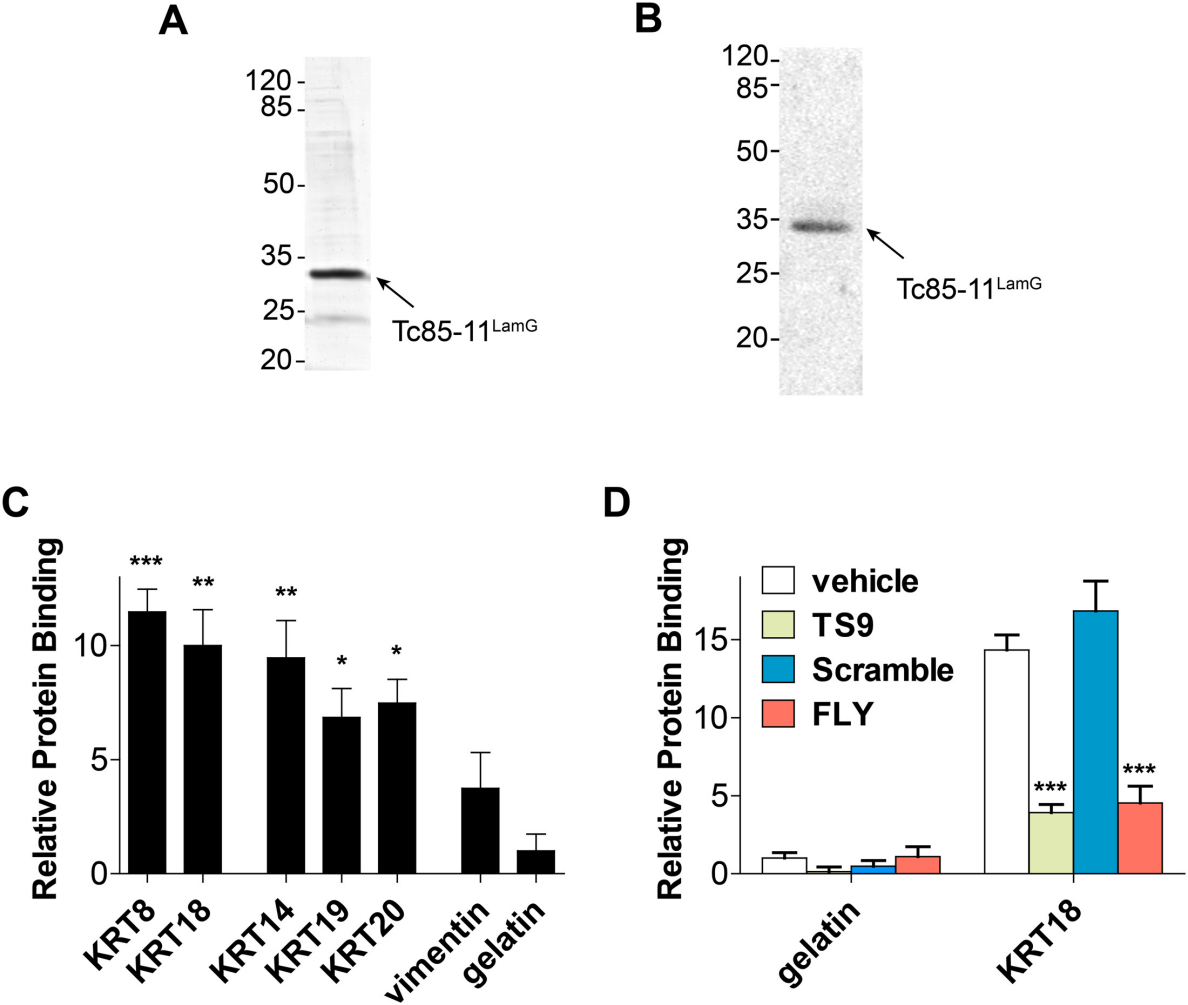
Tc85-11^LamG^ protein binds to cytokeratin. **(A)** Purified Tc85-11^LamG^ protein analyzed by SDS-PAGE stained with Coomassie blue and **(B)** immunoreactivity with an anti-gp85/TS monoclonal antibody. **(C and D)** Binding of Tc85-11^LamG^ protein to intermediate filament proteins. **(D)** Peptide competition assay. Effect of synthetic peptides scramble, TS9 and FLY on Tc85-11^LamG^ binding to KRT18 protein. The results were normalized by the negative control gelatin. Mean ± SEM of a representative experiment performed in triplicate are shown (* p<0.05, ** p<0.01***, p<0.001; one way ANOVA [**C**] or two way ANOVA [**D**]).

To confirm that the LamG domain of gp85/TS binds to cytokeratins, as suggested by the presence of the two conserved peptide motifs (TS9 and FLY), we produced in *E*. *coli* as recombinant protein the LamG domain of Tc85-11 (denominated Tc85-11^LamG^) (Fig 5A). The purified protein was recognized by an anti-gp85 mAb (Fig 5B). Similarly to phage TS9, the Tc85-11^LamG^ protein binds to all immobilized cytokeratins that we tested (Fig 5C). The binding was specific to cytokeratins and no interaction was observed with gelatin. To determine whether peptides TS9 and FLY play a role in LamG binding to cytokeratin, we performed competition assays using KRT18 as ligand. The binding of Tc85-11^LamG^ to KRT18 could be inhibited by the synthetic peptides FLY and TS9 but not by the scramble control peptide (Fig 5D). In different experiments (N = 3) peptide TS9 inhibited the binding of Tc85-11^LamG^ to KRT18 by 81% and peptide FLY by 70%; the combination of both peptides did not results in significant increase in the inhibitory levels. These results corroborate the data suggesting that peptides TS9 and FLY comprise a non-linear conformational binding site for cytokeratins.

### Peptide TS9 inhibits cell adhesion and reduces cell infection by *T*. *cruzi*

**Fig 6:**
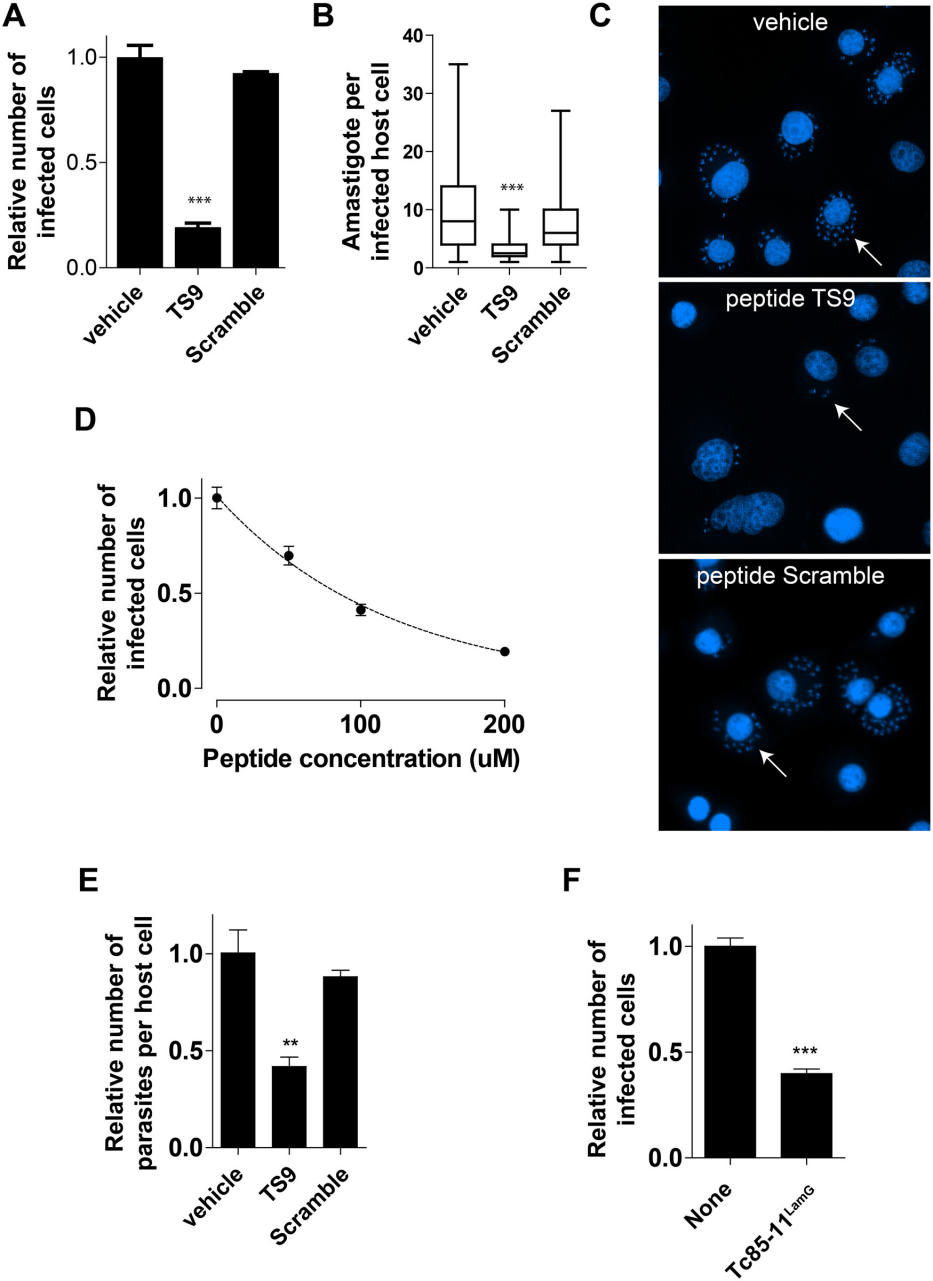
Peptide TS9 inhibits parasite cell adhesion and invasion. **(A and B)** Effect of peptide TS9 or the control scramble peptide (200 μM) on LLC-MK_2_ host cell infection by *T*. *cruzi*. In **(A)** the number of infected cells is indicated. Values are expressed as relative the number of infected cells (vehicle-only treatment values were set to 1). Mean ± SEM of a representative experiment performed in triplicate are shown. In **(B)** the number of parasites per infected host cells is shown. Data are presented in box plots in which the boxes define the 25th and 75th percentiles, with a line at a median and whiskers defining the maximum and minimum values of experiments performed in triplicate (*** p<0.001, one-way ANOVA). **(C)** Representative pictures of microscope fields used for the quantification of parasite invasion. Cells were stained with DAPI. The arrows indicate infected cells with amastigotes in the cytoplasm. (**D**) Dose dependence effect of peptide TS9 on LLC-MK_2_ host cell infection by the parasite. (**E**) Infection assay performed at 4°C to prevent parasite from entering host cells in the presence or absence of peptides TS9 or scramble (200 μM). (**F**) Effect of Tc85-11^LamG^ protein on parasite host cell infection.

The gp85/TS family members have long been associated with cell adhesion and invasion by our group [5,10,13] and others [26–28]. Having shown that the Tc85-11^LamG^ domain and the gp85/TS derived peptide TS9 bind to host cells and interact with cytokeratins, we tested whether they could modulate parasite cell infection. Epithelial LLC-MK_2_ cells were pre-incubated with synthetic peptide TS9 (200 μM) for 15 minutes before the addition of infectious trypomastigote forms to the cell culture. After 2 hours of infection, cells were washed to remove extracellular parasites, and further cultured for 48 hours. Cells were then fixed and the number of infected cells and parasites (amastigotes) per infected cells quantified. Peptide TS9 reduced *T*. *cruzi* infection by 78% (Fig 6A). It also reduced the number of parasites per cell by 69% (Fig 6B and 6C). Additionally, cell infection was reduced in a dose dependent manner (Fig 6D). Treatment of host cells and parasites with vehicle alone or with the scrambled version of peptide TS9 at the same concentration had no effect on parasite cell infection. To determine if the reduction in infection was due to a toxic effect of the peptide, cells were incubated with peptide TS9 (200 μM) for the same length of times used in the infection assays (2h for trypomastigotes or 48h for LLC-MK_2_ cells); no noticeable effect on cell viability was observed, suggesting that the peptide by itself is not toxic to either cell type (S2 Fig).

To assess the effect of peptide TS9 on *T*. *cruzi* cell adhesion, we repeated the infection assay in a lower temperature. By performing the infection assay at 4°C, a condition that allows for parasites to adhere but prevent them from entering the cell, we observed that peptide TS9 reduced by 58% the number of parasites attached to cells (Fig 6E). No effect was observed with the control scramble peptide. These results suggest that peptide TS9 affects *T*. *cruzi* adhesion to cells and, consequentially, reduces cell infection.

### The gp85/trans-sialidase LamG domain inhibits *T*. *cruzi* infection

Having shown that a peptide derived from the LamG domain of gp85/TS modulates cell invasion, we next determined the effect of this domain in the process. LLC-MK_2_ cells and trypomastigotes were incubated in the presence or absence of Tc85-11^LamG^ recombinant protein (6.5 μM) for 2h. Cells were then washed and cultured for 24h. There was a reduction of 60% in the number of infected cells by the Tc85-11^LamG^ protein (Fig 6F), confirming the importance of this domain in the parasite cell infection process.

**Fig 7:**
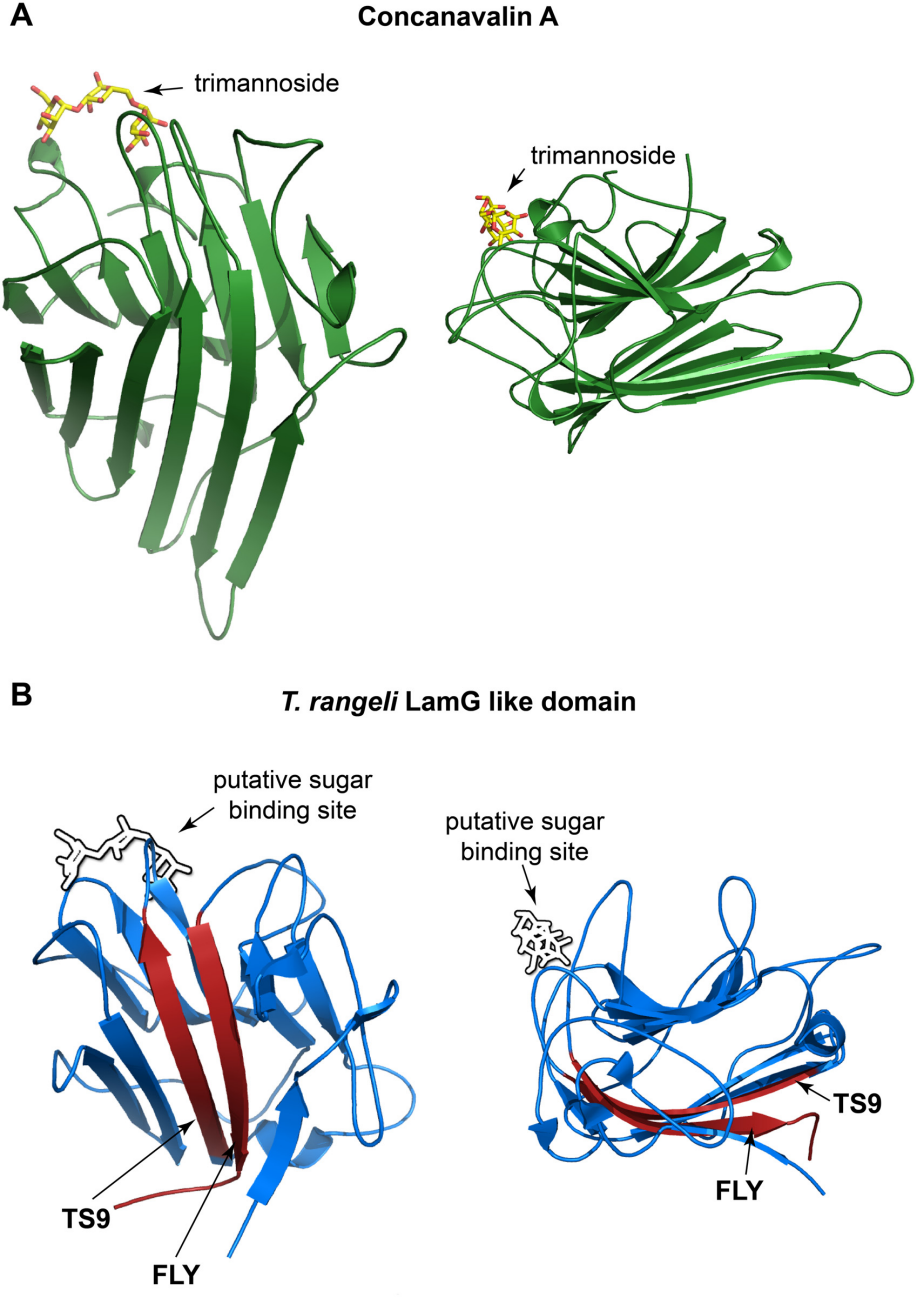
gp85/TS LamG domain putative binding sites. Ribbon diagrams representing protein structures. In (**A**), the 3D structure of concanavalin-A (PDB ID 1CVN chain A) with a bound trimannoside molecule is shown. In (**B**), the tertiary structure of the LamG domain from the *T*. *rangeli* sialidase (1WCS, amino acids 426–624) is shown. Arrows indicate the conserved peptides TS9 and FLY constituting the possible cytokeratin binding (red); and a putative carbohydrate binding site identified by structural analogy to the concanavalin-A molecule.

Of note, the LamG fold found in gp85/TS proteins is also present in the lectin concanavalin-A (protein family, pfam13385 concanavalin A-like lectin/glucanases superfamily). Concanavalin-A binds carbohydrates containing α-D-mannosyl and α-D-glucosyl groups and it is tempting to contemplate the possibility that the LamG domains of gp85/TS family members also have lectin like activity. Because some members of the gp85/TS family have the capacity to transfer sialic acids between carbohydrates chains, it is enticing to ponder whether the LamG domain of the gp85/TS proteins could bind to carbohydrates and facilitate this process. We thus analyzed the structures of concanavalin-A bound to a trimannoside molecule and the LamG domain of the *T*. *rangeli* trans-sialidase. By comparison of the 3D structures, the trimannoside binding site in concanavalin-A does not overlap with the antiparallel β-sheets cytokeratin binding site comprised of peptides TS9 and FLY (Fig 7). Moreover, the cytokeratin binding site has a relatively large surface area composed mostly of hydrophobic amino acids, compared with the smaller carbohydrates binding site in concanavalin-A, which is rich in hydrophilic residues.

## Discussion

Proteins belonging to the group II of the gp85/TS family have long been implicated in adhesion to cells and extracellular matrix components but the structural details of such interactions are still largely unknown. One of the difficulties to unveil the structural details of such interactions is the large number of proteins contained in these multigene families; having hundreds of individual members, it is often challenging to associate an individual member to its ligand. Besides, the characterization of each member separately is not practical. This task is particularly challenging in the case of *T*. *cruzi* where genetic tools like RNAi cannot be used [29]. Here we took a systematic approach to identify conserved peptide motifs shared by multiple members of the family and analyze them using bacteriophage and functional assays. This approach proved successful and led to the identification of the peptide TS9, a novel motif involved in parasite cell adhesion and invasion. Noteworthy is the fact that the identification of peptide TS9 further confirms previous results from our group and reinforces the knowledge that cytokeratins are an important ligands for *T*. *cruzi* adhesion and invasion [10,16,21]. It also highlights the importance in parasite biology of the LamG domain contained in all gp85/TS family members.

Cytokeratins are essential proteins for the cell structure and major constituents of intermediate filaments. Therefore, they are often viewed solely as intracellular proteins. However, it is of note that several studies have reported the ectopic expression of cytokeratins, such as in the extracellular milieu at the plasma membrane [16,17,30–33]. Studies from our group and others have emphasized the important role for extracellular cytokeratin expression in host-pathogen interactions [16,21,34,35]. And these reports pointing to an important role for extracellularly expressed cytokeratins are not limited to cell-parasite interactions. KRT8 expression on the surface of cancer cells has been associated with enhanced adhesion to fibronectin and the extracellular matrix [36,37]. Anti-KRT8 antibodies inhibit the urokinase-type plasminogen activator receptor binding and plasmin production by breast cancer cells, increasing tumor invasive potential [38].

It is worth mentioning that proteins belonging to other groups of filaments (microtubules and microfilaments) were also identified by affinity chromatography on peptide TS9. Whether they are receptors for the gp85/TS proteins remains to be investigated. It is known that amastigote forms do express proteins from the gp85/TS family and may, therefore, interact intracellularly with cytokeratins [27,39] and other cytoskeletal proteins. This interaction may be important for parasite proliferation in the cytoplasm. In fact, the knockdown of cytokeratin-18 has been shown to affect the parasite's ability to replicate inside host cells [40]. But because cells often express several cytokeratins and given the promiscuous interaction of gp85 with these different cytokeratin proteins, it is still difficult to assess the effects of a single gene knockdown on parasite cell invasion and proliferation.

The results of the inhibition assay in the present study suggest that peptide TS9 affects *T*. *cruzi* infection by interfering with the ability of the parasite to adhere to the cell surface, cytokeratin being one of the possible cell receptors involved in this process. These data corroborate previous findings from our group that have implicated the conserved peptide FLY and, therefore, the motif VTVxNVxLYNRPLN, in *T*. *cruzi* cell adhesion to cytokeratins/vimentin and invasion [16]. Nonetheless, we are puzzled by the fact that both peptides have sometime shown opposite effects in specific assays. For instance, peptide TS9 consistently inhibits parasite infection and blocks the interaction of LamG domain with cytokeratins; the peptide FLY on the other hand, also inhibits the binding of the LamG domain to cytokeratins but increases parasite cell infection [16]. One possible explanation for this conundrum is the fact that the peptide FLY has a direct effect on host cells, inducing cytokeratin remodeling and activation of ERK signaling cascades [21], which in turn might trigger alternative pathways utilized by the parasite to enter the cells. We do not know if peptide TS9 also affects host cells in the same manner. Notwithstanding the effect on cell infection, and in aggregate, our results point to an important role for these two conserved peptide motifs, the LamG domain and cytokeratins in parasite biology. Consequently, the mapping of two highly conserved cytokeratin binding peptides (TS9 and FLY) to the same binding site within the LamG domain is unlikely to be a coincidence but further studies will be necessary to unveil the molecular details of their interactions and how they operate in the process.

Noteworthy is the fact that the peptide TS9 overlaps with a previously described immunodominant epitope. Peptide TS9 (FTLVASVTI) shares the FTLV sequence found in two immunodominant peptides (ANYKFTLV and VNYDFTLV) derived from gp85/TS family members; these peptides are found within the same region of peptide TS9 [41,42]. Peptides ANYKFTLV and VNYDFTLV are dominant epitopes responsible for CD8^+^ T cells activation in mice and humans, with up to 40% of all CD8^+^ T cells responding to them. The host immune response represents an important selective pressure against parasite populations bearing these sequences. Despite the evolutionary pressure these peptides continue to be expressed by the parasite. Our results suggest that binding to cytokeratin may explain why these peptides have not changed over time, since they are necessary for parasite cell-host interaction.

Of note is our observation that gp85/TS proteins contain a LamG domain. The LamG domain or LNS domain (for Laminin-alpha, Neurexin and Sex hormone-binding globulin) is found in a large number of extracellular proteins. Examples of proteins containing LamG domains are laminins, collagens, agrin, neurexins, pentraxins, sex steroid binding protein (SBP/SHBG) and concanavalin-A, among others. It is notable the different functions and binding properties exhibited by LamG domains in these proteins. When present in adhesion molecules they mediate receptor binding, cell adhesion and migration [43]. The LamG domains found in the sex steroid binding protein SBP/SHBG bind small molecules such as estradiol [44]. On the other hand, the LamG domains present in pentraxins interact with a multitude of ligands derived from microbial pathogens and cellular debris produced by inflammatory processes [45]. Finally, in the concanavalin-A molecule, the LamG domain is lectin that binds to α-D-mannosyl and α-D-glucosyl residues. Whether the LamG domain in gp85/TS proteins bind carbohydrates and facilitates the transfer of sialic acids, it remains an open question. But if it does, as suggested by comparison with the concanavalin-A structure, it would probably constitute an entirely new binding site and possibly an interesting target for drug development.

Finally, this work outlines a novel systematic approach to study gene families found in *T*. *cruzi* and other parasites. By combining *in silico* analysis to reduce the complexity of a large family of proteins with phage display, we established a functional assay to screen the common peptide motifs shared by all members this family. This framework proved successful and led to the identification of a cell adhesion peptide, important for parasite cell invasion. Certainly, this approach can be applied to other gene families present in *T*. *cruzi* (or other organisms) such as mucin, mucin-associated proteins (MASP), dispersed gene family 1 (DGF-1) and GP63. Novel peptides relevant for host-parasite interaction, such as the cytokeratin binding peptide characterized in this study, may be identified and help to understand the molecular details of cell infection.

## Material and Methods

### Cells and *T*. *cruzi* culture

The epithelial LLC-MK_2_ cells (ATCC CCL-7) from *Macaca mulatta* (rhesus monkey) were used for *T*. *cruzi* culture and assays. Cells were maintained in Minimum Essential Media (MEM) supplemented with 10% fetal bovine serum (Life Technologies). *T*. *cruzi* Y strain was used for these studies and cultured and maintained as described [46].

### Peptide synthesis

Peptides were synthesized to our specifications with a minimum of 95% purity by Chinese Peptide Company (Hangzhou, China). The synthetic peptides used in this study were peptides TS9 (FTLVASVTI) and scramble (LTVIFATVS). The synthetic peptide FLY used for the competition assays had the original sequence described by Magdesian et al. (2001) (GKKPSVTVTNVFLYNRPLN) [16].

### Multiple alignment and identification of conserved motifs

The primary sequences of proteins belonging to all groups (I to VIII) of the gp85/TS were retrieved from TriTrypDB [22] using gene lists produced by Freitas et al. [11]. The sequences belonging to the gp85/TS group II were then aligned using Clustal Omega [47]. A grid profile was produced containing all alignment position and its respective conservation using UGENE [48]. The grid profile file contains the amino acid frequency (percentage) for each position in the alignment (S1 Table). For the selection of conserved regions in the gp85/TS proteins, the moving average of 6 up to 19 consecutive amino acid positions were calculated using the grid profile data. Logo sequence images were generated using WebLogo [49]. The sequences belonging to all gp85/TS groups were then aligned using Clustal Omega [47] to confirm whether the identified conserved motifs (TS1 to TS10) were also present in proteins belonging to other groups.

### Phage cloning

Complementary synthetic oligonucleotides (Exxtend, Brazil) encoding the selected gp85/TS derived peptides were annealed, cloned into the fUSE55 vector [50] and electroporated into *E*. *coli* cells (strain MC1061). After overnight culture, phage clones were produced, sequenced to verify the presence of insert and used to infect *E*. *coli* strain K91kan for phage large scale production. All phage were sequenced again to confirm the presence of insert and titrated by colony count (transducing units, TU) and qPhage (number of phage particles).

### Cell phage binding assay

The BRASIL method was used for phage-cell binding assays [23]. In brief, single cell suspensions (SCs) of LLC-MK_2_ cells produced by trypsin digestion followed by a 2h recovery period in MEM at 37°C in the incubator. SCs (10^6^/ml) were then incubated with individual phages (10^11^ phage particles in 200 μl of MEM 1% BSA) for 3h on ice. For peptide competition assays, cells and phage were incubated in the absence or presence of increasing peptide concentration. The admixture was centrifuged over the oil phase (dibuthylphtalate:cyclohexane [9/1] vol/vol) to separate bound from non-bound phages as previously described. Phage quantification was done by qPCR [51].

### Protein phage binding assay

Phage binding assays were performed as previously described [17]. Individual recombinant human cytokeratins (Cell Sciences), vimentin from bovine lens (Sigma-Aldrich Co) and gelatin from porcine skin (Sigma-Aldrich Co) were immobilized on wells of flat-bottom 96-well polystyrene plates (200 ng of protein in 50 μl of phosphate buffered saline solution [PBS]); overnight at 4°C). Wells were washed with PBS, blocked with PBS 1% BSA (1h) before 10^9^ TU of each individual phage in 50 μl of blocking buffer were added to the wells. After 2h, wells were washed 10 times with PBS and bound phage were recovered by bacterial infection and quantified by colony counting.

### Affinity chromatography

Synthetic peptide TS9 (1 mg) was conjugated to the agarose beads using the CarboxyLink Kit (Thermo Scientific) according to the manufacturer recommendations. LLC-MK_2_ cells were then lysed in PBS 2% Nonidet P-40, centrifuge at 10.000g for 30 minutes and the supernatant incubated with the peptide-coupled resin overnight at 4°C. After extensive wash with PBS and NaCl 1M, the resin was eluted sequentially with SDS 1% (1^st^ elution) and 8 M urea (2^nd^ elution). The eluted proteins were analyzed by SDS-PAGE and the gel stained with Coomassie brilliant blue G250 before processing for mass spectrometry. Protein bands with calculated molecular masses of 49, 53, 59 and 66 kDa were excised from the gel, sliced into small pieces, treated with dithiothreitol DTT (10 mM), iodoacetamide (100 mM) and then digested with sequencing grade trypsin (5 μg/ml) (Promega, WI, USA). The resulting peptides were then analyzed by HPLC-ESI-MS at the Analytical Center facility of the Chemistry Institute, University of São Paulo (São Paulo, Brazil). The proteins present in each gel fragment were identified by peptide fingerprinting using the MASCOT software [52].

### Western blot

Proteins were analyzed by SDS-PAGE and transferred to nitrocellulose membranes. The membranes were blocked for 1h at room temperature (RT) with Odyssey blocking buffer (LI-COR Biosciences) and incubated with anti-KRT18 antibody (1 μg/ml) (Invitrogen, Clone DC-10) followed by anti-mouse immunoglobulin-Ig antibodies conjugated with IRDye800 (LI-COR Biosciences). Immunoblot was then analyzed with the Odyssey Imaging System (LI-COR Biosciences).

### Peptide toxicity assay

LLC-MK_2_ cells (5x10^3^) were cultured in 100 μl MEM 10% FBS in 96 well polystyrene plate 24h prior to the experiment. Next day, media was replaced by MEM 2% FBS containing 1% DMSO and peptide TS9 or scramble (200 μM). Cells were then incubated for 2h or 48h. Trypomastigotes (3x10^6^) in 100 μl of MEM 2% FBS containing 1% DMSO and peptides TS9 or scramble (200 μM) were cultured for 2h. Cell viability was determined using the WST-1 reagent according to manufacturer’s recommendations (Roche).

### *T*. *cruzi* cell invasion assay

LLC-MK_2_ cells (3x10^4^) were cultured in 24 well polystyrene plate containing glass coverslips 24h prior to the experiment. For the peptide inhibition assay, increasing concentrations of peptides were pre-incubated with host cells in MEM 2% FBS containing 1% DMSO and after 15 minutes, trypomastigotes (3x10^6^) were added to the cells (final volume of 400 μl). After 2 hours infection, cells were washed 10 times with MEM 2% FBS to remove free trypomastigotes and cells were culture for 24 or 48h. For the adhesion assay, host cells and parasites were incubated at 4°C during the 2h infection period and then washed 10 times with MEM 2% FBS. The experiment with the recombinant Tc85-11^LamG^, cells and parasites were incubated with 6.5 μM of protein in MEM 2% FBS. Cells were then fixed and stained with DAPI and photographed with a Nikon epifluorescence microscope using a 20x objective. The number of infected cells was then quantified from at least 4 fields for each treatment and the number of parasites per infected cells was then calculated from at least 100 infected cells.

### Molecular modeling of Tc85-11

The amino acid sequence of the Tc85-11 protein were retrieved from GenBank (AAD13347.1) and used to identify conserved domains using the NCBI Conserved Domain software (CD-Search) [53]. A 3D structural model was then generated with using the Iterative Threading ASSEmbly Refinement algorithm (I-TASSER) at http://zhanglab.ccmb.med.umich.edu/I-TASSER/ [25]. The full-length model is constructed by iterative template fragment assembly simulations and by comparing the 3D models with a protein function database. The *Trypanosoma rangeli* trans-sialidase (PDB id: 1WCS) was used as an additional restraint template to generate the model. The best model generated had a template modelling score (TM-Score) of 0.50±0.15. For reference, A TM-score >0.5 indicates a model of correct topology (independent of protein length) and a TM-score <0.17 means a random similarity [54].

### Tc85-11^LamG^ domain cloning and expression

The DNA encoding the LamG domain of the Tc85-11 protein (accession number AAD13347, amino acids 475 to 677) was amplified by PCR using specific primers (forward GACCATATGCTGGTGGCGGTATTGTCAAAC and reverse GACGTCGACATTCAGTGGGCGGTTGTACAG), and cloned into pET21a vector using the *Nde* **I** and *Sal* **I** restriction enzymes. The plasmid was sequenced to confirm the presence of insert and absence of mutations. Recombinant Tc85-11^LamG^ protein was produced in *E*. *coli* Rosetta (DE3) pLysS by culturing in LB media until OD_600nm_ ~0.7 Abs and then induced with 0.8 mM isopropyl β-D-1-thiogalactopyranoside (IPTG) for 4h at 37°C. Cells were lysed in denaturing binding buffer (100 mM phosphate buffer pH 8.0, 10 mM Tris, 8M urea) and recombinant protein purified in Ni-NTA column (Qiagen). The resin was washed with denaturing washing buffer (100 mM phosphate buffer pH 6.3, 10 mM Tris, 8M urea) and bound protein eluted with denaturing elution buffer (100 mM phosphate buffer pH 3.5, 10 mM Tris, 8M urea). A 1 M Tris pH 8.0 solution was used to adjust the pH of the eluate. The recombinant protein was renatured by stepwise dialysis against PBS containing decreasing concentrations of urea (4, 2, 1 and 0.5 M urea over a period of 2h each). The final dialysis was against PBS overnight.

### Protein binding assay

Cytokeratins or vimentin were immobilized on 96-wells microtiter plates (200 ng in 50 μl of PBS) (4°C, overnight), blocked with Odyssey blocking buffer for 1h at 37°C and incubated with Tc85-11^LamG^ recombinant protein in PBS (60 μg/ml). Wells were then washed 3 times with PBS and bound protein detected using an anti-gp85 monoclonal antibody (G1G8, kindly provided by Dr. Eliciane Mattos), followed by anti-mouse immunoglobulin-Ig antibodies conjugated with IRDye680 (LI-COR Biosciences). Plates were then analyzed with the Odyssey Imaging System (LI-COR Biosciences). The monoclonal antibody G1G8 recognizes a neutralizing epitope (sequence TGETPLEPFGFCFGA) within the LamG domain of the Tc85-11 protein [14]. For the competition experiments, the Tc85-11^LamG^ recombinant protein in PBS (60 μg/ml) was incubated in the absence or presence of peptides (200 μM).

## Supporting Information

S1 Table Multiple sequence alignment grid profile generated for gp85/TS sequences group II. (XLS)

S2 Table Physicochemical properties for the TS peptides. (PDF)

S3 Table Proteins identified by affinity chromatography and mass spectrometry as possible ligands for peptide TS9. (PDF)

S1 Fig TS peptides conservation in the gp85/TS family. Representation in sequence logo format of the gp85/TS derived peptides in all groups (I to VIII) of the gp85/TS family. The letter size indicates amino acid conservation in each position and the color, whether amino acids are polar (green), hydrophobic (black), positively (blue) or negatively (red) charged.
(TIF)

S2 Fig Toxicity effect of peptides TS9 and scramble on *T*. *cruzi* and host cells. Trypomastigotes and LLC-MK_2_ cells were incubated with peptides TS9 or scramble (200 μM) for 2h (**A** and **B**) or 48h (**C**) and analyzed for cell viability. Mean ± SEM of a representative experiment performed in triplicate are shown (one way ANOVA).
(TIF)
