# Supplementary material for: Trypanosoma cruzi Binds to Cytokeratin through Conserved Peptide Motifs Found in the Laminin-G-Like Domain of the gp85/Trans-sialidase Proteins
Source: PLoS Negl Trop Dis. 2015 Sep 23;9(9):e0004099. doi: 10.1371/journal.pntd.0004099 (PMC4580646; doi:10.1371/journal.pntd.0004099)
Supplement: S2 Table — (PDF) [file pntd.0004099.s002.pdf]

Supplementary Table II – Hydrophobicity, pI and average charge of peptides at pH 7.4. The free energy of transfer from aqueous to apolar media was calculated according to the scale proposed by WIMLEY; WHITE (1996). Average charge in physiological pH and pI was calculated using the ideal model as described in TEIXEIRA; LUND; DA SILVA (2010).

Peptide	$\Delta G_{Hydrophobicity}$ (kcal/mol)	pI	Average charge in pH 7,4
**TS 1a**	1.71	4.0	-1.44
**TS 1b**	-0.45	5.5	-0.44
**TS 2**	0.23	10.0	1.56
**TS 3**	1.67	4.4	-1.45
**TS 4**	1.68	11.0	2.55
**TS 5**	-1.82	5.8	-0.45
**TS 6**	-1.20	5.1	-1.37
**TS 7**	3.57	4.5	-1.44
**TS 8**	-0.09	5.2	-0.44
**TS 9**	-1.28	5.5	-0.44
**TS 10**	2.12	6.0	-0.45
**FLY**	-0.18	8.7	0.55

TEIXEIRA, A. A. R.; LUND, M.; DA SILVA, F. L. B. Fast Proton Titration Scheme for Multiscale Modeling of Protein Solutions. **J. Chem. Theory Comput.**, v. 6, n. 10, p. 3259–3266, 2010.

WIMLEY, W. C.; WHITE, S. H. Experimentally determined hydrophobicity scale for proteins at membrane interfaces. **Nature structural biology**, v. 3, n. 10, p. 842–848, out. 1996.
