# Supplementary material for: Trypanosoma cruzi Binds to Cytokeratin through Conserved Peptide Motifs Found in the Laminin-G-Like Domain of the gp85/Trans-sialidase Proteins
Source: PLoS Negl Trop Dis. 2015 Sep 23;9(9):e0004099. doi: 10.1371/journal.pntd.0004099 (PMC4580646; doi:10.1371/journal.pntd.0004099)
Supplement: S3 Table — (PDF) [file pntd.0004099.s003.pdf]

Supplementary Table III. Proteins identified by affinity chromatography and mass spectrometry as possible ligands for peptide TS9. The proteins identified in each gel fragment (a, b, c and d) are shown. The table includes the score generated by the protein identification software (Mascot)(higher numbers indicate optimum separation of signal peaks from noise peaks and therefore higher confidence), gene name, protein name and annotated protein molecular mass.

a Apparent Mass = 49 kDa

Score	Gene Name	Name	Mass (kDa)
809	KRT18	Keratin, type I cytoskeletal 18	48
535	ACTB	Actin, cytoplasmic 1	42
534	KRT8	Keratin, type II cytoskeletal 8	54

b Apparent Mass = 53 kDa

Score	Gene Name	Name	Mass (kDa)
723	KRT8	Keratin, type II cytoskeletal 8	54
377	EEF1A1	Elongation factor 1-alpha 1	50

c Apparent Mass = 59 kDa

Score	Gene Name	Name	Mass (kDa)
451	KRT1	Keratin, type II cytoskeletal 1	65
439	TUBB	Tubulin beta chain	50
404	ATP5A1	ATP synthase subunit alpha, mitochondrial	60

d Apparent Mass = 66 kDa

Score	Gene Name	Name	Mass (kDa)
683	HSP90AB1	Heat shock protein HSP 90-beta	83*
510	HSP90AA1	Heat shock protein HSP 90-alpha	85

* The proteins also has 68 kDa isoform annotated (uniprot entry Q58FF7)
