# Supplementary figures and images for: Trypanosoma cruzi Binds to Cytokeratin through Conserved Peptide Motifs Found in the Laminin-G-Like Domain of the gp85/Trans-sialidase Proteins
Source: PLoS Negl Trop Dis. 2015 Sep 23;9(9):e0004099. doi: 10.1371/journal.pntd.0004099 (PMC4580646; doi:10.1371/journal.pntd.0004099)

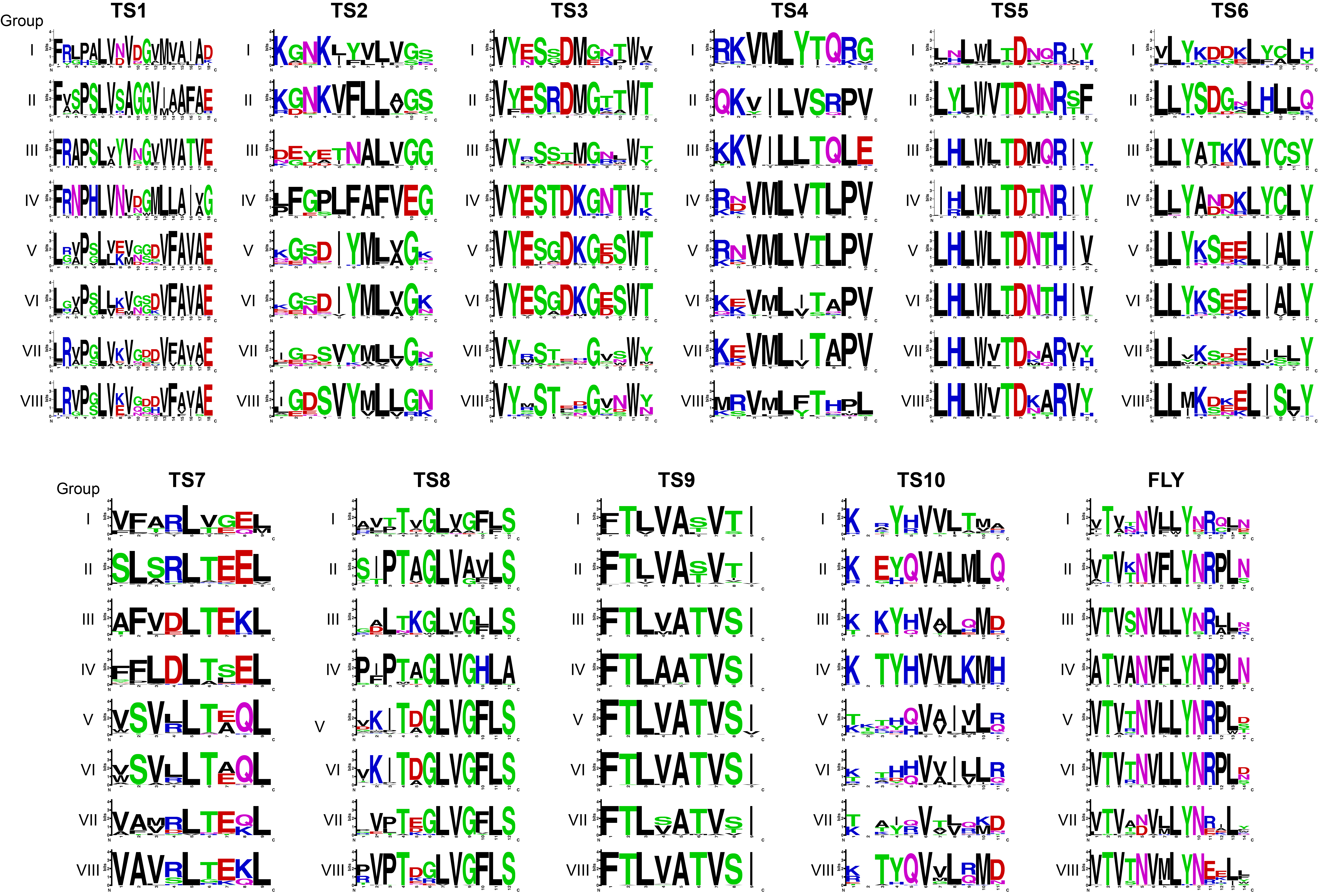

Supplement: S1 Fig — Representation in sequence logo format of the gp85/TS derived peptides in all groups (I to VIII) of the gp85/TS family. The letter size indicates amino acid conservation in each position and the color, whether amino acids are polar (green), hydrophobic (black), positively (blue) or negatively (red) charged. (TIF) [file pntd.0004099.s004.tif]

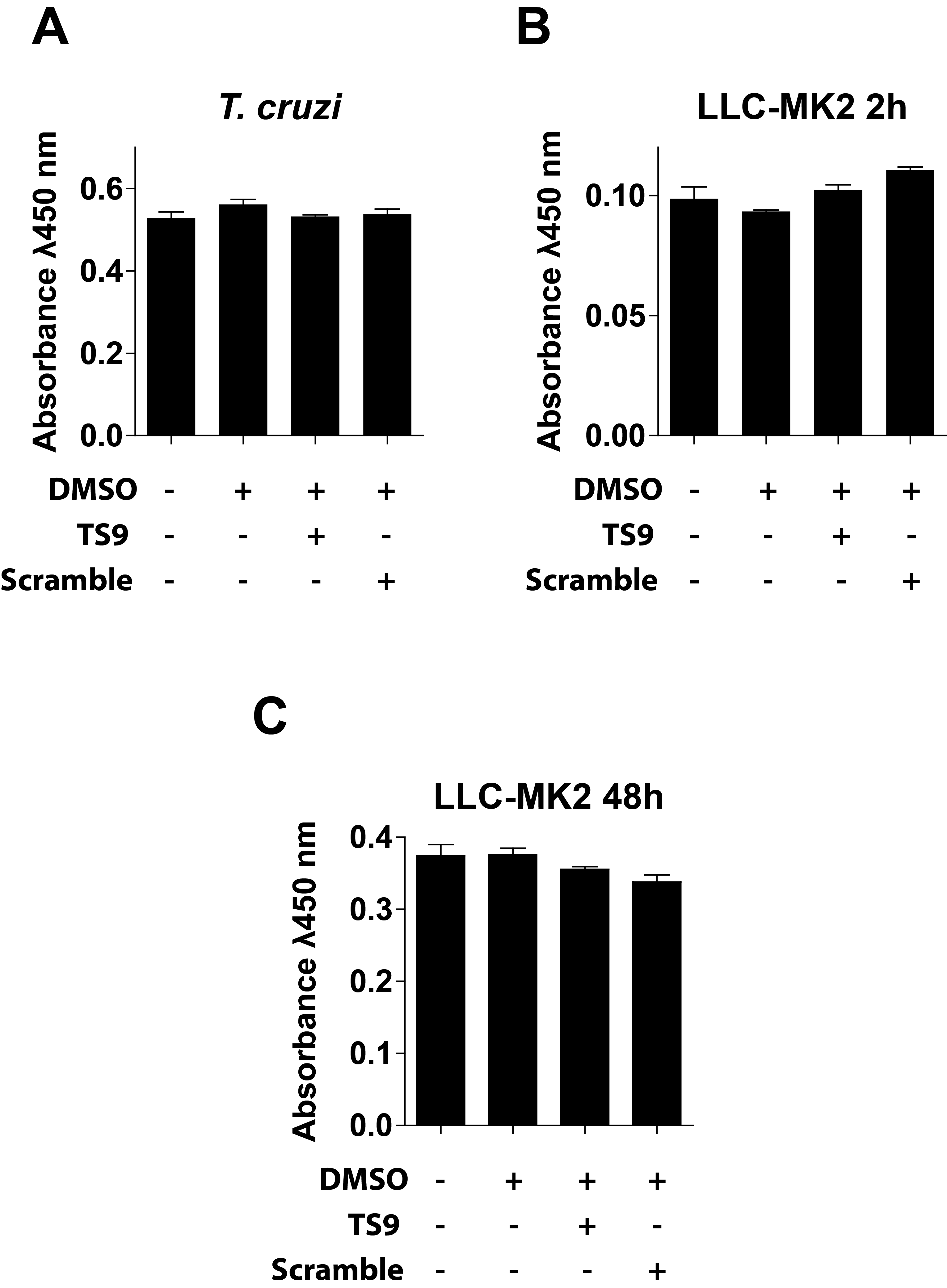

Supplement: S2 Fig — Trypomastigotes and LLC-MK2 cells were incubated with peptides TS9 or scramble (200 μM) for 2h (A and B) or 48h (C) and analyzed for cell viability. Mean ± SEM of a representative experiment performed in triplicate are shown (one way ANOVA). (TIF) [file pntd.0004099.s005.tif]
